# Gamma-Irradiated Starch/Chitosan Composite Particles for the Adsorption of *Neospora caninum* Membrane Proteins as a Potential Vaccine Delivery Platform

**DOI:** 10.3390/polym18192447

**Published:** 2026-10-08

**Authors:** María Paula Palma-Calvo, Laria Rodríguez-Quesada, Randall Chacón-Cerdas, Manuel Vilanova, Ricardo Starbird-Perez

**Affiliations:** 1 Master’s Program in Medical Devices Engineering, Instituto Tecnológico de Costa Rica, Cartago 159-7050, Costa Rica; 2 Marie Sklodowska-Curie Fellowship Programme, International Atomic Energy Agency (IAEA), 1220 Vienna, Austria; 3 Centro de Investigación en Biotecnología, Escuela de Biología, Instituto Tecnológico de Costa Rica, Cartago 159-7050, Costa Rica; 4 Centro de Investigación en Servicios Químicos y Microbiológicos (CEQIATEC), Escuela de Química, Instituto Tecnológico de Costa Rica, Cartago 159-7050, Costa Rica; 5 Departamento de Física, Facultad de Ciencias Exactas y Naturales, Universidad Nacional, Heredia 86-3000, Costa Rica; 6 Instituto de Investigação e Inovação em Saúde, Universidade do Porto, 4200-135 Porto, Portugal; 7 Instituto de Ciências Biomédicas de Abel Salazar (ICBAS), Universidade do Porto, 4050-313 Porto, Portugal

**Keywords:** protein adsorption, nasal drug delivery, subunit vaccine, mucosal immunization, antigen delivery

## Abstract

Gamma irradiation offers a simple and reagent-free strategy for tailoring the physicochemical properties of starch for the development of functional biomaterials, although its application in protein delivery systems remains largely unexplored. In this study, starch was irradiated at doses between 50 and 500 kGy and combined with chitosan to produce composite particles for the adsorption of *Neospora caninum* membrane proteins (NcMP), with the aim of developing a potential intranasal vaccine delivery platform. Irradiation induced progressive oxidation and depolymerization of starch, as evidenced by Fourier transform infrared (FTIR) analysis, increased apparent amylose content, reduced molecular weight and hydrodynamic diameter, and shifted the zeta potential to −13.4 mV, leading to the selection of starch irradiated at 300 kGy for particle preparation. The resulting starch–chitosan particles exhibited spherical morphology and, following protein adsorption, formed a protein-associated nanosystem with a hydrodynamic diameter of 105.7 nm and a zeta potential of −18.8 mV. Protein association was confirmed by fluorescence co-localization and an adsorption efficiency of 34%. These findings demonstrate that gamma irradiation is an effective approach for engineering starch-based composite particles with physicochemical characteristics suitable for protein association and intranasal delivery, supporting their potential as a versatile platform for protein subunit vaccine formulations.

## 1. Introduction

Modern vaccine development has progressively shifted toward the use of defined protein antigens in subunit vaccines because of their improved safety profile compared with whole-pathogen vaccines [1]. However, their susceptibility to degradation and relatively low immunogenicity often limit their effectiveness when administered alone, necessitating the development of delivery systems capable of preserving antigen integrity and enhancing immune responses [2,3]. Delivery systems based on nano- and microparticles have emerged as promising strategies to address these limitations by protecting antigens, promoting their interaction with immune cells, and prolonging antigen availability at the administration site [4,5,6].

Nasal immunization has the advantage of inducing both local and systemic immune responses, characterized by the production of secretory IgA (sIgA) and the activation of tissue-resident memory T cells (TRM) [5], both of which are critical for protection against pathogens entering through mucosal surfaces [6,7]. In addition, the nasal-associated lymphoid tissue (NALT) promotes immune responses at distant mucosal sites, including the oral, pulmonary, vaginal, and rectal mucosae, providing broad mucosal protection [7,8,9]. Achieving these immunological advantages depends on carrier systems with physicochemical properties that promote antigen retention, interaction with the mucosal epithelium, and efficient uptake by immune cells [10,11].

Among the various physicochemical modification strategies available for starch, gamma irradiation has emerged as an effective approach [12]. Gamma irradiation induces cleavage of glycosidic bonds and oxidation reactions, leading to reductions in molecular weight and the formation of carbonyl and carboxyl groups [13,14]. These structural and chemical modifications alter the physicochemical and functional properties of starch, expanding its potential for pharmaceutical and biomedical applications, including its use in functional carrier systems [15]. Beyond its effects on starch structure, gamma irradiation is also considered a green modification technology because it enables controlled structural modification without the use of chemical reagents, thereby reducing the need for conventional chemical modification processes [16].

Considering these requirements, biopolymers have emerged as attractive platforms for antigen delivery because they can be engineered to enable particle formation, antigen association, and tunable interfacial properties [17,18]. Among the wide range of available biopolymers, starch and chitosan offer complementary functionalities that are particularly relevant for potential mucosal vaccine applications. Starch is especially attractive as a carrier material because it is abundant and biodegradable, and its physicochemical properties can be readily modified through various approaches to tailoring its molecular weight, surface charge, and interaction capacity [19,20,21]. Structural modifications, such as chain scission and oxidation, can alter the physicochemical properties of starch and promote the exposure or formation of negatively charged functional groups [13,14]. Chitosan, in turn, is a cationic polysaccharide whose protonatable amino groups can promote electrostatic interactions with negatively charged biomolecules and polymeric surfaces [22,23]. Consequently, the combination of starch and chitosan can promote polymer–polymer association through electrostatic interactions between anionic groups in starch and protonated amino groups in chitosan, as previously demonstrated in starch–chitosan polyplexes with tunable particle size and surface charge [24,25]. Previous studies have also demonstrated the use of starch-based nanoparticles for antigen delivery [26] and chitosan-based particulate systems for nasal vaccine delivery [22,23], highlighting the versatility of these natural polymers as delivery platforms and their potential for tailoring particle size, surface charge, and cargo association according to the intended application.

Novel veterinary parasite vaccines are particularly important for reducing reliance on drug treatments for the control of parasitic diseases and for preventing the emergence of drug-resistant parasite phenotypes [27]. *Neospora caninum* is an intracellular parasite that causes abortion and reproductive losses in cattle, resulting in significant economic losses to the livestock industry [28]. Despite its importance, there is currently no commercially available vaccine that provides effective protection against neosporosis [29]. Although intranasal immunization with *N. caninum* membrane proteins (NcMP) has demonstrated promising protective immune responses [30], the successful development of NcMP-based vaccines requires delivery systems capable of preserving antigen integrity while providing the physicochemical properties necessary for efficient intranasal delivery [31]. To improve antigen delivery in NcMP-based vaccines, this study investigates the development and physicochemical characterization of composite particles prepared from gamma-irradiated starch and chitosan, along with a membrane protein antigen derived from *N. caninum*, as a candidate particulate antigen delivery system for intranasal vaccination.

## 2. Materials and Methods

### 2.1. Materials and Reagents

Starch from corn (S4126; CAS 9005-25-8; lot MKCN1761), a regular, unmodified corn starch containing approximately 27% amylose and 73% amylopectin, was purchased from Sigma-Aldrich (St. Louis, MO, USA). According to the manufacturer, the product is stored at room temperature and has a specified moisture content below 15%. Low-molecular-weight chitosan (75–85% degree of deacetylation; product no. 448869; lot BCCP3419; molecular weight 50–190 kDa based on viscosity; viscosity 20–300 cP) was purchased from Sigma-Aldrich (St. Louis, MO, USA). Soybean oil was purchased from a local commercial supplier in Costa Rica. According to the product label, the oil contains approximately 60.7% polyunsaturated, 25.0% monounsaturated, and 14.3% saturated fatty acids by mass, with linoleic acid (omega-6) and alpha-linolenic acid (omega-3) contents of approximately 53.6% and 7.1%, respectively.

Dimethyl sulfoxide (DMSO), ethanol (>95%), acetic acid, sodium acetate, sodium bicarbonate, calcium chloride (CaCl_2_), acetone, phosphate-buffered saline (PBS), Span 80, Calcofluor White LRP, and 4-chloro-7-nitrobenzofurazan (NBD-Cl) were purchased from Sigma-Aldrich (St. Louis, MO, USA). Ultrapure water (18.2 MΩ·cm at 25 °C) was obtained using a Milli-Q water purification system (Millipore, Burlington, MA, USA).

### 2.2. Gamma Irradiation of Starch

To obtain gamma-irradiated starch for subsequent particle synthesis, starch dispersions were prepared by suspending 500 mg of starch in 25 mL of DMSO and gently vortexed. The dispersion was heated in boiling water for 1 min, vortexed vigorously, and heated for an additional 15 min with intermittent stirring.

The starch dispersions were transferred to screw-cap glass vials (6 cm × 1.5 cm) and irradiated in a Co-60 gamma cell irradiator (Ob-Servo Ignis, IZOTOP, Budapest, Hungary) following a previously reported protocol [32]. Samples were irradiated at doses ranging from 50 to 500 kGy. The overall uncertainty of the absorbed dose rate was 2.4% (95% confidence level).

Following irradiation, starch was recovered by ethanol precipitation. Aliquots (2 mL) of the irradiated starch dispersions were mixed with 4 mL of 95% (*v*/*v*) ethanol under continuous vortexing, followed by the addition of a further 8 mL of ethanol. The mixtures were allowed to stand for 15 min and were then centrifuged at 2000× *g* for 5 min (Sorvall LEGEND X1R, Thermo Scientific, Waltham, MA, USA). The supernatant was discarded, and the resulting pellets were air-dried for 10 min prior to physicochemical characterization.

### 2.3. Physicochemical Characterization of Gamma-Irradiated Starch

Fourier transform infrared (FTIR) spectroscopy was performed using a Nicolet Apex FTIR Spectrometer (Thermo Scientific, Madison, WI, USA) to identify changes in functional groups. Approximately 2 mg of dried starch was placed directly onto the diamond ATR crystal, and spectra were acquired in absorbance mode over the range of 4000–600 cm^−1^ using 32 scans at a resolution of 4 cm^−1^.

Hydrodynamic particle size, zeta potential, and molecular weight were determined for each irradiation dose using a Zetasizer Nano ZS (Malvern Panalytical, Malvern, UK). All measurements were performed in purified water at 25 °C and at a scattering angle of 173°. Values were calculated from measurements performed in triplicate.

Amylose content was estimated using an iodine-based spectrophotometric assay according to the manufacturer’s instructions (Megazyme Amylose/Amylopectin Assay Kit, K-AMYL, Bray, Ireland) [33].

### 2.4. Preparation of Starch–Chitosan Composite Particles

I-St–Chitosan (I-St–Chit) composite particles were prepared using starch irradiated at 300 kGy (I-St) following a modified water-in-oil emulsion dehydration method adapted from Chacón-Cerdas et al. [34]. The aqueous phase was prepared by dissolving 0.1% (*w*/*v*) chitosan in 2% (*v*/*v*) acetic acid under magnetic stirring. After complete dissolution, 0.1% (*w*/*v*) I-St was incorporated under continuous stirring until a homogeneous dispersion was obtained. The oil phase was prepared by adding 1125 µL of Span 80 to 100 mL of soybean oil and homogenizing the mixture under mechanical stirring (BDC6015, Caframo, Georgian Bluffs, ON, Canada) at 1000 rpm for 5 min. Next, 25 mL of the aqueous phase was added dropwise into the oil phase under continuous stirring for 5 min, followed by the addition of 2.5 mL of 2 M CaCl_2_ as the crosslinking agent. After an additional 5 min, 125 mL of acetone was added to dehydrate the particles. The suspension was stirred at 500 rpm for an additional 5 min at room temperature. Finally, the resulting particles were purified by three consecutive acetone washes, each followed by centrifugation at 2000× *g* for 10 min, and dried overnight at 34 °C.

### 2.5. Physicochemical Characterization of I-St–Chit Composite Particles

Particle morphology was evaluated by scanning electron microscopy (SEM) (TM-3000, Hitachi, Tokyo, Japan) at an accelerating voltage of 15 kV after mounting the samples on aluminum stubs with double-sided carbon adhesive tape. FTIR spectra of chitosan, I-St, and I-St–Chit particles were acquired under the same experimental conditions described for irradiated starch. Hydrodynamic particle size and zeta potential of I-St–Chit particles were determined using a Zetasizer Nano ZS (Malvern Panalytical, Malvern, UK).

### 2.6. Extraction of N. caninum Membrane Proteins (NcMP)

*Neospora caninum* membrane proteins (NcMP) were extracted from free tachyzoites of the Nc1 isolate (ATCC^®^ 50843) propagated in VERO cell cultures, following a previously described methodology [35] with modifications. Briefly, tachyzoites were resuspended in PBS containing 0.75% Triton X-114, using 3 mL of lysis solution per 5 × 10^8^ parasites, and incubated on ice for 10 min. Samples were centrifuged at 10,000× *g* for 30 min at 4 °C, and the supernatants were incubated at 30 °C for 3 min to promote phase separation. The samples were subsequently centrifuged at 10,000× *g* for 30 min at 4 °C and then at 1000× *g* for 3 min at room temperature. The NcMP-containing hydrophobic phase was recovered and precipitated with four volumes of absolute ethanol, followed by vortexing for 15 s and incubation on ice for 1 h. Samples were centrifuged at 12,000× *g* for 20 min at 4 °C, and the resulting pellets were air-dried, resuspended in PBS, and stored at −20 °C. Protein concentration was determined using the Pierce™ BCA Protein Assay Kit (Thermo Fisher Scientific, Rockford, IL, USA) according to the manufacturer’s instructions (Figure S2 and Table S1), and protein integrity was evaluated by SDS-PAGE.

### 2.7. Protein Adsorption onto I-St–Chit Particles

I-St–Chit particles were suspended, and lyophilized NcMP were reconstituted separately in 50 mM sodium acetate buffer (pH 4.0) at 1 mg/mL. Both suspensions were mixed at a 1:1 (*v*/*v*) ratio in 1.5 mL polypropylene microcentrifuge tubes and incubated overnight under gentle orbital agitation (100 rpm; Digisystem DSR-2800D, New Taipei City, Taiwan) at 20 °C in the dark. Following incubation, the samples were centrifuged at 12,000× *g* for 10 min (MiniSpin^®^, Eppendorf, Hamburg, Germany), and the resulting pellets were washed three times with 2 mL of acetate buffer (pH 4.0). Finally, the pellets were resuspended in PBS (pH 7.2) for subsequent physicochemical characterization. Hydrodynamic particle size and zeta potential of I-St–Chit particles following NcMP adsorption were subsequently determined using a Zetasizer Nano ZS (Malvern Panalytical, Malvern, UK).

### 2.8. Fluorescence Validation of Protein Adsorption

I-St–Chit particles were labeled with Calcofluor White, whereas NcMP were labeled with NBD-Cl. Excess fluorochrome was removed by centrifugal filtration using Amicon^®^ Ultra-2.0, 10 kDa MWCO (Merck, Darmstadt, Germany). Protein adsorption was performed under the conditions described above. Co-localization of the labeled particles and proteins was evaluated by confocal fluorescence microscopy using a BioTek Cytation™ C10 Confocal Imaging Reader (Agilent Technologies, Santa Clara, CA, USA) equipped with DAPI (Ex 365 nm/Em 435 nm) and GFP (Ex 470 nm/Em 535 nm) filter sets. Merged fluorescence images were generated using Gen5™ software (version 3.12, Agilent Technologies, Santa Clara, CA, USA).

### 2.9. Protein Adsorption Efficiency

Protein concentration was determined using the QuantiPro™ BCA Assay Kit (Sigma-Aldrich, St. Louis, MO, USA). Briefly, 150 µL of the NcMP solution prior to adsorption and 150 µL of the resuspended I-St–Chit particles following NcMP adsorption were each mixed separately with 150 µL of BCA working reagent in a 96-well microplate and incubated at 60 °C for 1 h, protected from light. Absorbance was measured at 562 nm, and protein concentrations were determined using a calibration curve (Figure S3). The adsorption efficiency was calculated as the percentage of the initial NcMP concentration recovered in association with the particles after washing, according to Equation (1):

Adsorption efficiency (%) = (C_associated_/C_initial_) × 100 (1)

where C_associated_ is the NcMP concentration measured in the final I-St–Chit particle suspension and C_initial_ is the initial NcMP concentration before adsorption.

## 3. Results and Discussion

### 3.1. Characterization of Irradiated Starch Dispersion

**Figure 1:**
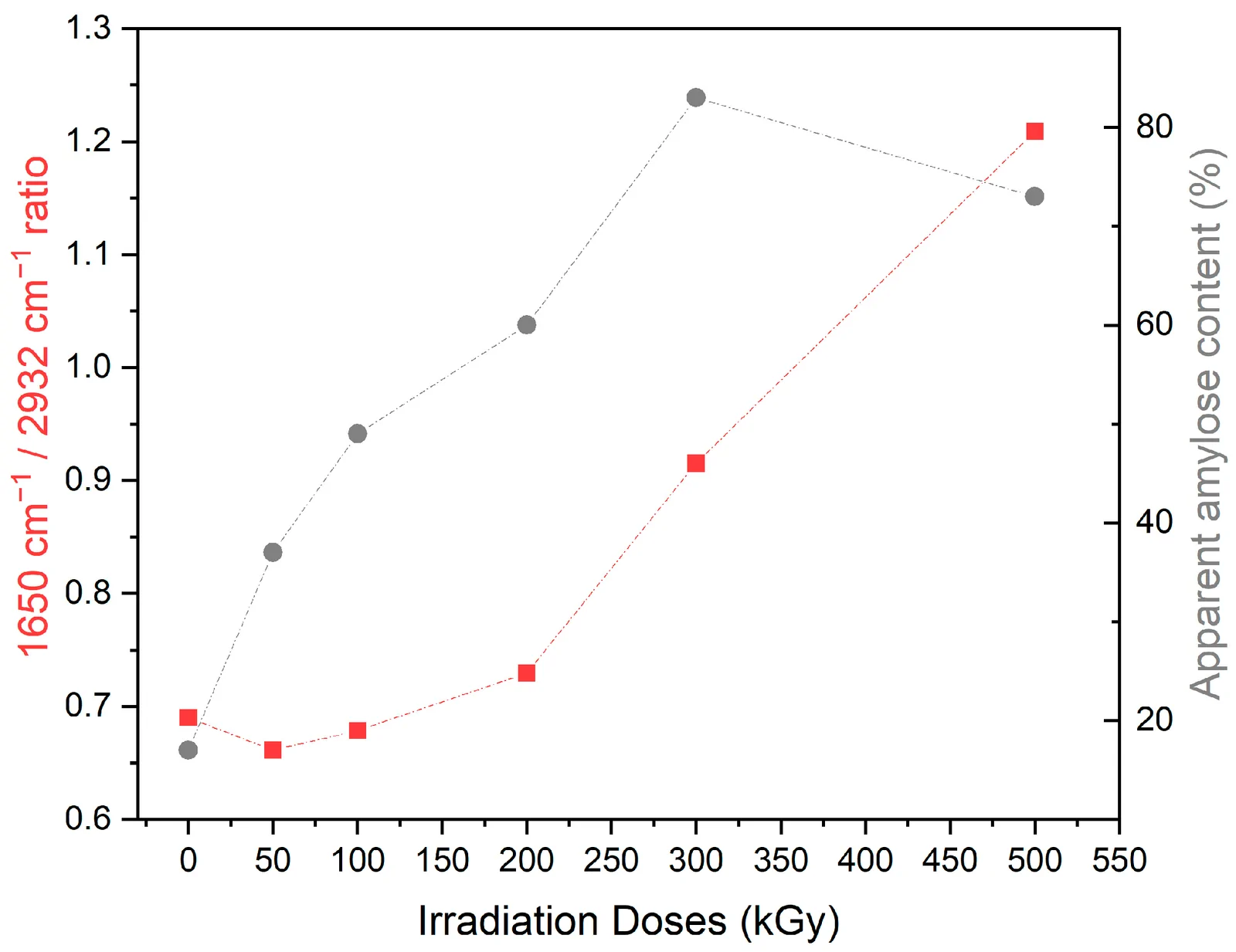
FTIR absorbance ratio (1650 cm^−1^/2932 cm^−1^) (red squares) and apparent amylose content (gray circles) of irradiated starch at different gamma doses.

Gamma irradiation induced clear chemical modifications in starch, as evidenced by FTIR analysis (Figure 1 and Figure S1). A progressive decrease in the intensity of the band around 3400 cm^−1^, assigned to O–H stretching, suggests reduced intramolecular hydrogen bonding and disruption of the semi-crystalline structure. In parallel, the gradual increase in the band near 1650 cm^−1^, observed from 200 kGy onward, is attributed to the formation of carboxyl-containing groups generated during the oxidative degradation of the polysaccharide backbone [36,37]. The progressive increase in the 1650 cm^−1^/2932 cm^−1^ absorbance ratio further confirmed the incorporation of oxidized functional groups, consistent with gamma-induced cleavage of glycosidic bonds (Figure 1) [26].

In agreement with the FTIR results, an apparent increase in amylose content was observed at doses ≥ 100 kGy (Figure 1). This behavior is consistent with the irradiation-induced depolymerization of amylopectin into shorter, less branched glucan chains that are quantified as amylose by the iodine-based colorimetric assay, rather than a true increase in native amylose. Similar behavior has been reported for irradiated starches, in which amylopectin fragmentation results in higher apparent amylose values [38,39,40]. Therefore, these findings suggest that gamma irradiation promotes the oxidative modification and depolymerization of starch, providing the basis for the observed changes in its physicochemical properties.

**Figure 2:**
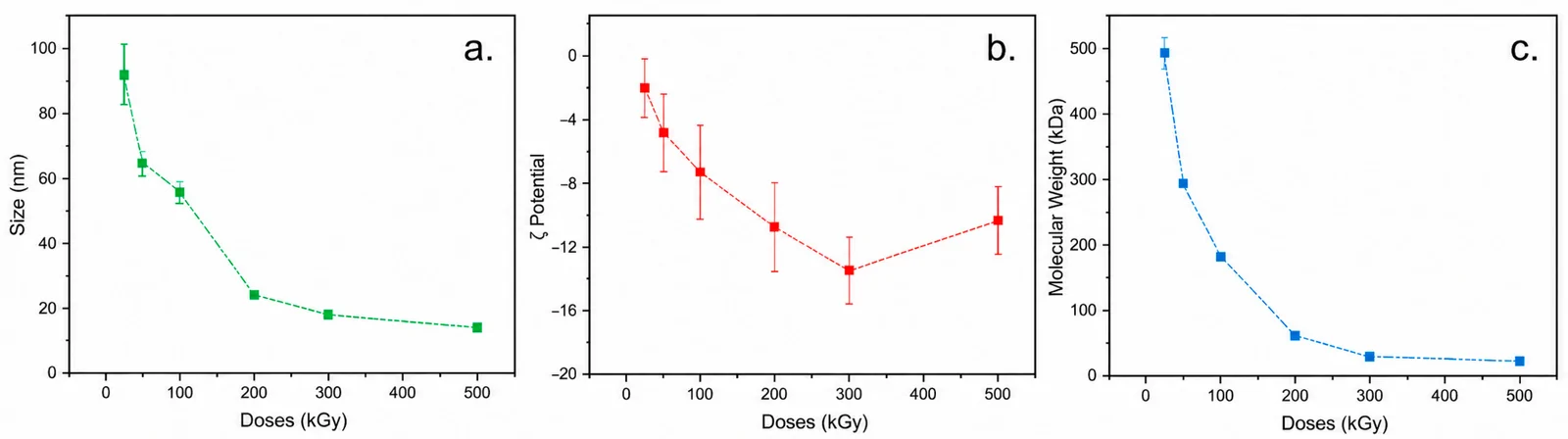
Effect of gamma irradiation on starch physicochemical properties. (**a**) Hydrodynamic diameter (nm) (green squares). (**b**) Zeta potential (mV) (red squares). (**c**) Molecular weight (kDa) of irradiated starch samples from 25 to 500 kGy (blue squares).

The chemical modifications induced by gamma irradiation were reflected in the physicochemical properties of starch particles (Figure 2). The zeta potential shifted toward more negative values, reaching −13.4 ± 3.93 mV at 300 kGy, reflecting the incorporation of oxidized functional groups, such as carboxyl moieties, into the polymer backbone [17,41]. This more negative surface charge enhanced electrostatic repulsion between particles, thereby improving colloidal stability while promoting subsequent electrostatic interactions with chitosan during composite particle formation [42]. In parallel, irradiation-induced chain scission was accompanied by a progressive reduction in hydrodynamic diameter, from approximately 100 nm to 13.5 nm, together with a decrease in molecular weight from 493 kDa to 26.5 kDa. These trends are consistent with extensive depolymerization of the starch chains, yielding lower-molecular-weight structures with a reduced hydrodynamic size [17,43].

Among the evaluated doses, 300 kGy provided the most favorable balance between oxidative modification and structural integrity. Although higher irradiation doses promoted further reductions in hydrodynamic diameter and molecular weight, the slight decrease in zeta potential observed at 500 kGy suggests that additional irradiation did not produce further beneficial changes in the measured physicochemical properties. Overall, the combination of increased oxidation, higher negative surface charge, reduced hydrodynamic diameter, and lower molecular weight supported the selection of starch irradiated at 300 kGy (I-St) as the optimal substrate for subsequent composite particle formation and protein adsorption.

### 3.2. Particle Formation and Protein Adsorption

**Figure 3:**
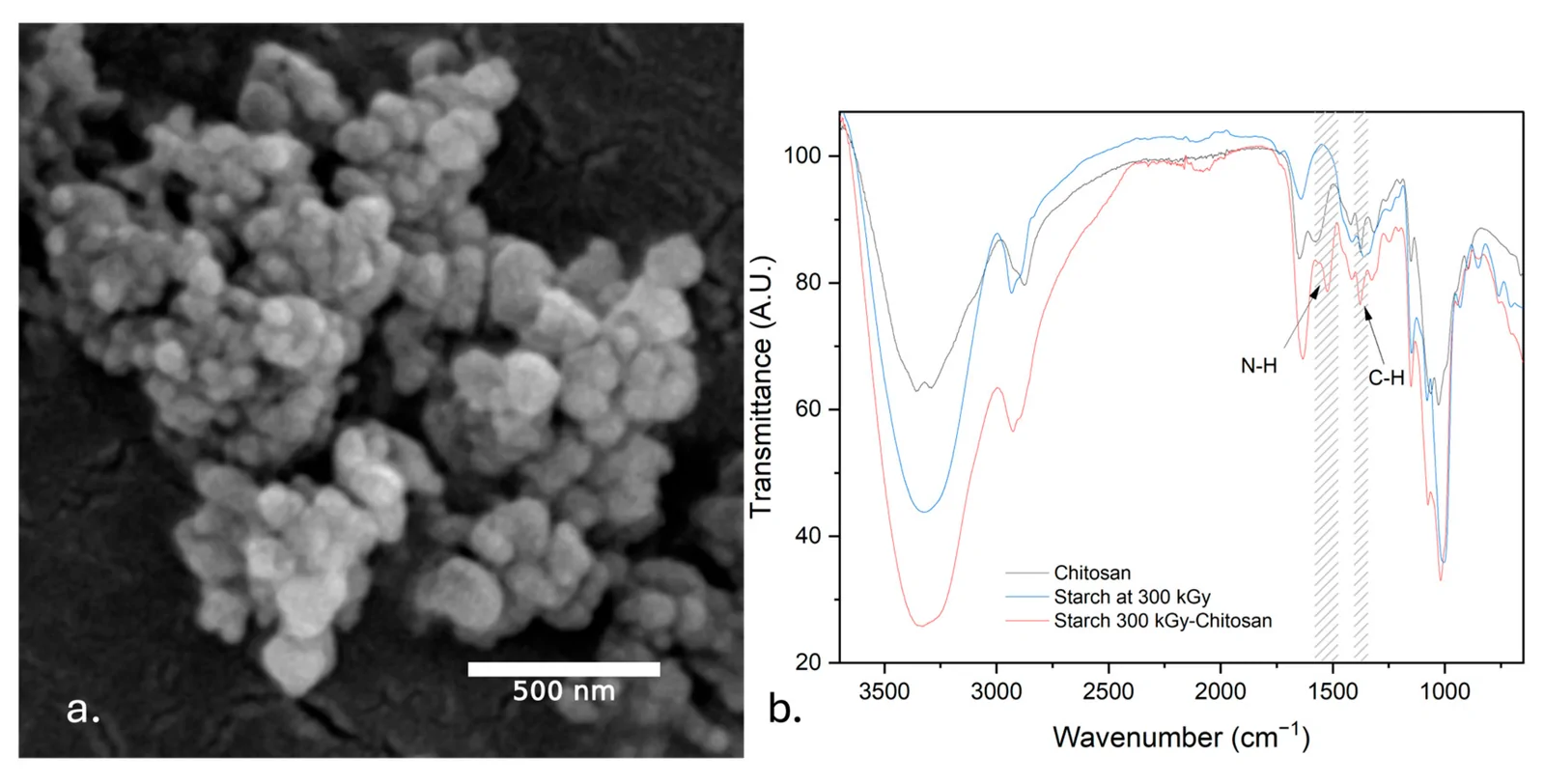
Characterization of I-St–Chit particles. (**a**) SEM micrographs of I-St–Chit particles at 10,000× magnification. (**b**) FTIR spectra of chitosan (black), starch (blue), and I-St–Chitosan particles (red).

The obtained particles prepared from gamma-irradiated starch and chitosan (I-St–Chit) exhibited a predominantly spherical morphology (Figure 3a), characteristic of systems formed through the dehydration of emulsion droplets during particle solidification [34]. The incorporation of CaCl_2_ as a crosslinking agent also influenced particle morphology by promoting stabilization of the polymer network through ionic interactions with negatively charged polysaccharide groups [34]. In addition, the intrinsic positive charge of chitosan, arising from protonated amino groups under acidic conditions, promoted electrostatic interactions with the oxidized starch chains, thereby contributing to polymer complexation and the formation of stable spherical particles [44,45].

FTIR analysis provided additional evidence of the intermolecular interactions established within the I-St–Chit system, as demonstrated by the broad band around 3300 cm^−1^ (Figure 3b), which was assigned to overlapping O–H and N–H stretching vibrations. This band is consistent with hydrogen bonding between starch and chitosan chains [46]. Together, hydrogen bonding and electrostatic interactions between oxidized functional groups on starch and protonated amino groups of chitosan contribute to the formation of a cohesive polymer network [44].

A highly positive surface charge (+43.3 mV ± 7.98 mV) was observed for the I-St–Chit particles, indicating strong electrostatic stabilization of the dispersion and suggesting that protonated amino groups from chitosan dominate the particle interface. Following association with NcMP, which exhibited a zeta potential of (−8.81 ± 0.01) mV at pH 7, the zeta potential shifted to (−18.8 ± 0.1) mV. This change reflects a modification of the interfacial composition of the particles, where protein-associated functional groups contribute to the external surface properties of the system. The observed change in surface charge reflects changes in the interfacial composition upon protein association, consistent with the dynamic behavior of nanoparticle interfaces interacting with biomolecules [47]. Together, these results support the formation of a protein–particle complex mediated by surface interactions.

**Figure 4:**
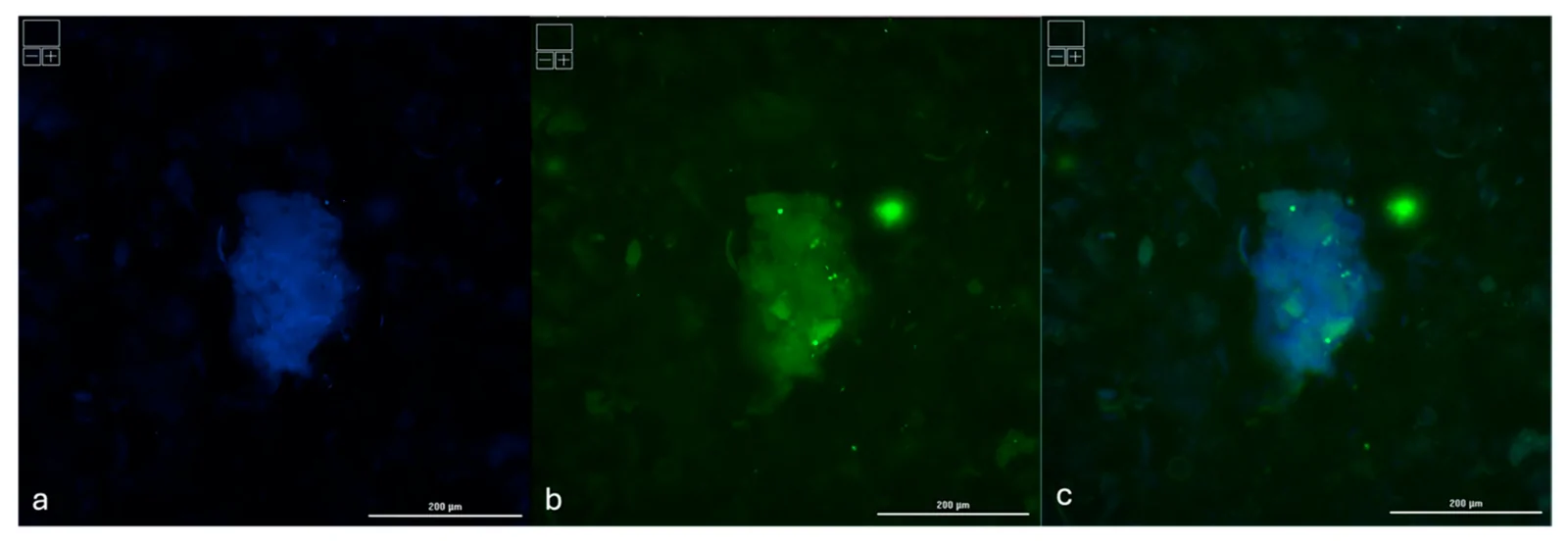
Confocal fluorescence microscopy images of I-St–Chit particles associated with NcMP. (**a**) Blue fluorescence (Calcofluor staining) indicates the I-St–Chit particles (DAPI filter, Ex 365 nm/Em 435 nm). (**b**) Green fluorescence (NBD labeling) indicates NcMP (GFP filter, Ex 470 nm/Em 535 nm). (**c**) Merged image showing the spatial co-localization of the blue and green fluorescence signals, supporting the association of NcMP with the particles.

Co-localization of the polysaccharide matrix and NcMP signals was observed in the merged fluorescence images (Figure 4c), supporting the association of NcMP with the composite particles. This observation is consistent with the changes in surface charge observed following protein incorporation and provides complementary evidence of protein–particle interactions [48]. BCA analysis indicated that approximately 34% of the initial NcMP concentration was recovered in association with the composite particles after the washing procedure (Table S2). This result provides complementary evidence of NcMP association with the particles; however, potential contribution of the I-St–Chit matrix to the BCA signal cannot be completely excluded. The observed protein recovery is consistent with the variability reported for chitosan-based delivery systems governed by non-covalent interactions, where loading efficiency depends on protein properties and particle surface characteristics [49,50,51]. These results suggest that protein association is governed by physicochemical compatibility and may be further optimized through formulation parameters.

The resulting protein-associated particles exhibited a hydrodynamic diameter of 105.7 nm, which falls within the size range considered optimal for intranasal delivery [52]. Nanoparticles close to 100 nm have been shown to exhibit prolonged residence time on the nasal epithelium and enhanced transport through the olfactory and trigeminal pathways compared with larger particles, while particles smaller than 200 nm generally show improved interaction with the nasal mucosa and more efficient transport across mucus barriers [52,53]. Together with the observed surface charge properties, these characteristics support the potential applicability of the system as a mucosal delivery platform.

Overall, these results demonstrate that particles prepared from gamma-irradiated starch and chitosan are capable of associating with biologically relevant proteins. The resulting system exhibits physicochemical characteristics suitable for mucosal delivery, particularly in terms of surface charge, particle size, and protein interaction, supporting its potential as a platform for intranasal vaccine delivery. Further studies are required to evaluate its biological performance and ability to induce protective immune responses.

## 4. Conclusions

This study demonstrated that gamma-irradiated starch can be successfully combined with chitosan to produce composite nanoparticles capable of associating with *Neospora caninum* membrane proteins. Starch irradiated at 300 kGy exhibited physicochemical properties suitable for particle formation, while the resulting protein-associated system displayed a hydrodynamic diameter of 105.7 nm, a negative surface charge, and an adsorption efficiency of 34%. Collectively, these findings demonstrate the feasibility of using gamma-irradiated starch–chitosan particles as a protein delivery platform with potential application in intranasal veterinary vaccine formulations. Future studies will focus on further evaluating these particles for intranasal vaccine delivery applications. In particular, the assessment of antigen release under physiological conditions, mucoadhesive properties, nasal retention, cellular uptake, and immune responses will be necessary to establish their suitability for mucosal vaccine delivery.

## Data Availability

The original contributions presented in this study are included in the article/Supplementary Materials. Further inquiries can be directed to the corresponding author.

## Supplementary Materials

The following supporting information can be downloaded at https://www.mdpi.com/article/10.3390/polym18192447/s1, Figure S1. FTIR spectra of irradiated starch with increasing gamma doses (0, 50, 100, 200, 300, 500 kGy); Figure S2. BCA calibration curve used for the quantification of *Neospora caninum* membrane proteins (NcMP); Table S1. Protein concentration of the hydrophobic (NcMP), hydrophilic, and interphase fractions obtained from *Neospora caninum*; Figure S3. BCA calibration curve used for the quantification of protein associated with I-St–Chit particles; Table S2. Protein concentration of the initial NcMP solution and protein-associated I-St–Chit particles used to calculate protein adsorption efficiency.

## Abbreviations

The following abbreviations are used in this manuscript:

DLS	Dynamic light scattering
FTIR	Fourier transform infrared spectroscopy
SEM	Scanning electron microscopy
I-St	Gamma-irradiated starch at 300 kGy
I-St–Chit	Composite particles prepared from starch irradiated at 300 kGy and chitosan
NcMP	*Neospora caninum* membrane proteins
PBS	Phosphate-buffered saline
