# Supplementary material for: Gamma-Irradiated Starch/Chitosan Composite Particles for the Adsorption of Neospora caninum Membrane Proteins as a Potential Vaccine Delivery Platform
Source: Polymers (Basel). 2026 Oct 8;18(19):2447. doi: 10.3390/polym18192447 (PMC13667077; doi:10.3390/polym18192447)
Supplement: Supplementary file 1 [file polymers-18-02447-s001.zip › polymers-4534557-supplementary.pdf]

Gamma-irradiated starch/chitosan composite particles for adsorption of *Neospora caninum* membrane proteins as a potential intranasal vaccine delivery platform

María Paula Palma-Calvo[1,2,5*], Laria Rodríguez-Quesada[3,4], Randall Chacón-Cerdas[5], Manuel Vilanova[6,7] and Ricardo Starbird-Pérez[3]

Supplementary Material

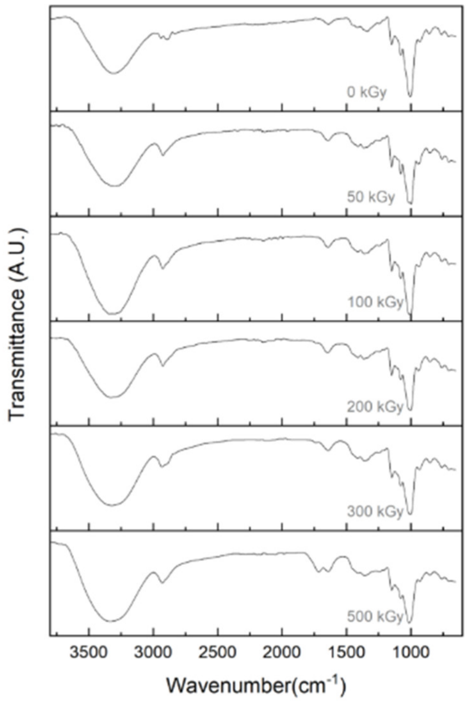

Figure S1. FTIR spectra of irradiated starch with increasing gamma doses (0, 50, 100, 200, 300, 500 kGy).

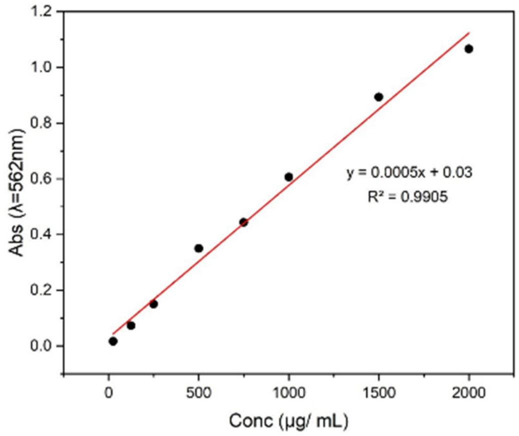

Figure S2. BCA calibration curve used for the quantification of *Neospora caninum* membrane proteins (NcMP)

Table S1. Protein concentration of the hydrophobic (NcMP), hydrophilic, and interphase fractions obtained from Neospora caninum

Samples	Absorption (λ=562nm)	Concentration (µg/ mL)
NcMP	0.841	1622
Hydrophilic proteins	0.336	612
Interphase	0.7645	1469

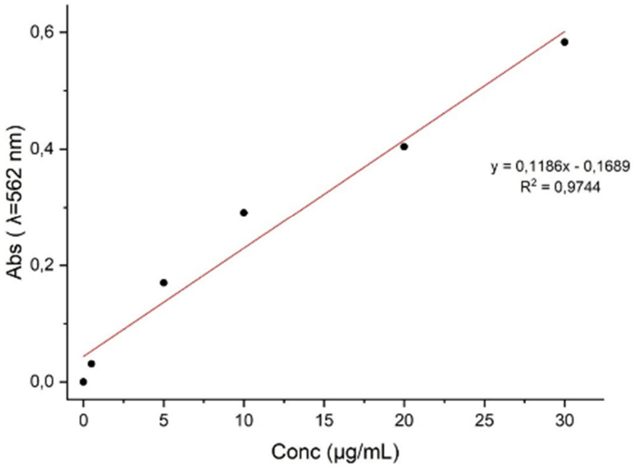

Figure S3. BCA calibration curve used for the quantification of protein associated with I-St–Chit particles

Table S2. Protein concentration of the initial NcMP solution and protein-associated I-St–Chit particles used to calculate protein adsorption efficiency.

Samples	Absorption (λ=562nm)	Concentration (µg/ mL)
NcMP	1.296	12.36
St-Chit-NcMP	0.337	4.27
